# Alternative lengthening of telomeres is dependent on *RAD52* and *RAD51* gene function in the budding yeast *Naumovozyma castellii*

**DOI:** 10.1093/g3journal/jkag155

**Published:** 2026-06-16

**Authors:** Humberto Itriago, Marita Cohn

**Affiliations:** Department of Biology, Genetics Group, Lund University, Lund SE-223 62, Sweden; Department of Biology, Genetics Group, Lund University, Lund SE-223 62, Sweden

**Keywords:** telomere, telomerase-deficient, alternative lengthening of telomeres, homologous recombination, budding yeast, *Naumovozyma castellii*

## Abstract

Telomerase is a specialized ribonucleoprotein enzyme responsible for elongation of telomeres, the DNA ends of eukaryotic chromosomes. In the absence of telomerase activity, telomeres progressively shorten, eventually triggering a DNA damage response that leads the cell to halt the cell cycle, a process known as replicative senescence. In budding yeast, the telomerase core enzyme consists of Est2, the catalytic subunit that acts as a reverse transcriptase, in complex with the noncoding telomerase RNA template (*TLC1*). *Saccharomyces cerevisiae* cells lacking any of these 2 components exhibit a severe growth crisis, which only few cells manage to survive by activating alternative lengthening of telomeres (ALT) pathways. In contrast, telomerase-deficient strains of the budding yeast *Naumovozyma castellii* can bypass senescence, by rapidly activating an ALT mechanism that allows the cells to maintain short and stable telomeres. The process involves a spreading of the telomere-adjacent TelKO element, leading to homogenization of the subtelomeric regions. In this study, we investigated the genetic requirements of the ALT mechanism in *N. castellii*, by analyzing deletion mutants of several genes involved in DNA recombination. By applying a quantitative growth assay, we demonstrate that the establishment of the ALT cells requires *RAD52* and *RAD51* gene function, while the *RAD50* and *RAD59* genes are not essential. This implies that the *N. castellii* ALT mechanism is dependent on homologous recombination with a similar genetic basis as the type I pathway in *S. cerevisiae* and shows that subtelomeric regions are playing a key role in the effective activation of ALT.

## Introduction

Linear chromosomes of eukaryotic cells require the presence of functional nucleoprotein terminal structures, known as telomeres, to protect the integrity of the genome. Telomeres are highly dynamic and regulated structures constituted by tandem TG-rich repeats and a group of proteins that bind these repeats with high affinity and specificity. The telomeric proteins prevent chromosomal end-to-end fusions, degradation, and the recognition of the telomeric DNA as damaged DNA by the DNA repair machinery, preventing chromosomal instability (de Lange 2009; Sfeir and de Lange 2012). Limitations of the canonical DNA replication machinery and processing of the telomeres by nucleolytic degradation during replication leads to progressive shortening of the telomeres with each replication cycle (de Lange 2009).

Telomere shortening prevents the binding of telomeric proteins, eventually leading cells to a type of growth arrest known as replicative senescence (Martinez-Fernandez et al. 2025). The enzyme telomerase, a specialized ribonucleoprotein complex with reverse transcriptase activity, prevents progressive telomere shortening by adding telomeric repeats to the single-stranded 3′ ends of the chromosomes using its internal telomerase RNA molecule as a template (Greider and Blackburn 1985; Cohn and Blackburn 1995; Vega et al. 2003). Because human somatic cells do not express sufficient telomerase, their telomeres shorten with each replication cycle. This progressive shortening of the telomeres functions as a biological clock promoting the cellular aging process after a number of cell divisions and induces replicative senescence (Blackburn et al. 2015). Cancer cells reverse and avoid further replicative senescence mainly by upregulating telomerase expression; however, some cancer cells develop telomerase-independent mechanisms for telomere maintenance, like the homologous recombination-based alternative lengthening of telomeres (ALT) mechanism (Sugarman et al. 2019).

In budding yeast, the in vitro telomerase activity requires 2 main structural components: the catalytic subunit (Est2) that acts as a reverse transcriptase and the noncoding telomerase RNA (*TLC1*) that functions as the template for the DNA synthesis (Singer and Gottschling 1994; Lingner et al. 1997). *Saccharomyces cerevisiae* cells deficient in either of these components will lack telomerase activity and therefore exhibit telomere shortening in each cell division, eventually leading to a replicative senescence. The senescence phenotypes are manifested as a severe decrease in cell proliferation and smaller colonies with rough edges appearing 50 to 100 generations after the loss of telomerase activity (Lundblad and Blackburn 1993; Chen et al. 2001). However, a low number of cells manage to survive the lethal consequences of telomere shortening, due to activation of ALT pathways (Lundblad and Blackburn 1993). *S. cerevisiae* survivor cells have been classified as type I and type II, based on the ALT pathway used by the cells (Lundblad and Blackburn 1993; Chen et al. 2001). Both types utilize a noncanonical type of homology-directed repair mechanism known as break-induced replication (BIR) that requires Rad52, the main recombination protein in yeast, and a nonessential DNA polymerase δ subunit, Pol32 (Lundblad and Blackburn 1993; Lydeard et al. 2007). Type I survivors keep short telomeric regions but recombine the Y’ subtelomeric elements located proximal to the telomere region by utilizing a Rad51-dependent BIR mechanism with Rad52 acting as a mediator for Rad51 filament formation and strand invasion (Chen et al. 2001; McEachern and Haber 2006; Symington et al. 2014). Type II survivors maintain long telomeres by utilizing Rad51-independent BIR, where strand annealing is promoted by the Rad52–Rad59 complex by short-sequence homology between the telomeric repeats. This mechanism additionally requires the MRX (Mre11, Rad50, and Xrs2) complex and Sgs1 (Teng et al. 2000; Chen et al. 2001; McEachern and Haber 2006). While type I and type II cells utilize different homology regions, recent data suggest that the generation of survivors may be enabled by a concerted action of the pathways, with early short telomeres being rescued by the Rad51-dependent pathway and later maintained by the Rad51-independent pathway (Kockler et al. 2021).

Telomere maintenance in ALT cancer cells is carried out by a mechanism involving homologous recombination, having similar genetic requirements as *S. cerevisiae* ALT cells (Pickett and Reddel 2015). An alternative method of recombinational telomere extension has been described for the budding yeast *Kluyveromyces lactis* (McEachern and Blackburn 1996). For this species, telomerase-deficient mutants use a “roll-and-spread” mechanism, where telomeric sequences on extrachromosomal circular (ECC) DNA molecules are used as the template for elongation of a shortened telomere. Subsequent elongation of other telomeres is then performed by BIR, using the elongated telomere as a copy template (Natarajan and McEachern 2002; Groff-Vindman et al. 2005).

The budding yeast *Naumovozyma castellii* is a useful model organism in telomere studies and possesses canonical short and regular 8-mer telomeric repeats (TCTGGGTG) and a highly processive telomerase activity (Cohn and Blackburn 1995; Karademir Andersson and Cohn 2017). *N. castellii* double-stranded telomeric DNA has a mean length of 320 ± 30 bp, and the single-stranded 3′ overhang shows a length range of 14 to 200 nt (Cohn et al. 1998; Fridholm et al. 2013; Itriago et al. 2022). The regular repeats have enabled the determination of the minimal binding sites of the telomeric proteins Rap1 and Cdc13, as well as their role in protection from exonucleolytic degradation (Runnberg et al. 2017; Runnberg et al. 2019). In the absence of telomerase activity, *N. castellii* cells rapidly activate an ALT mechanism that allows for the sustained growth of the cells, without exhibiting any of the severe phenotypes often associated with replicative senescence (Cohn et al. 2019; Jaiswal et al. 2025). Intriguingly, a specific subtelomeric sequence, the TelKO element, was shown to spread to all chromosome ends, leading to a homogenization of the subtelomeric regions (Jaiswal et al. 2025). Although sporadically occurring massive telomere lengthening events can be seen in terminal restriction fragment (TRF) assays, the telomeres of populations of *N. castellii* ALT cells are mainly maintained as stable shortened structures, which may indicate a tight balance between extension and trimming events.

In this study, we investigated the early events in the establishment of *N. castellii* ALT cells by using a quantitative senescence assay. Our results demonstrate that telomerase-deficient strains (*est2Δ* and *tlc1^−^*) bypass replicative senescence by rapidly activating the ALT mechanism. To investigate the genetic requirements of the ALT pathway, we created double mutants where telomerase deficiency was combined with deletions of genes involved in recombinational pathways. The *RAD52* gene was previously identified and characterized in *N. castellii*, and here we describe the identification of the gene homologs of *RAD50*, *RAD51*, and *RAD59* (Itriago et al. 2023). The senescence assay results of the double knockouts (double-KOs) show that the survival of telomerase-deficient cells is dependent on *RAD52* and *RAD51* gene function, indicating that the ALT pathway is dependent on Rad52–Rad51-mediated homologous recombination. Notably, we found no evidence supporting the requirement for *RAD50* or *RAD59*, suggesting that a Rad51-independent BIR mechanism is not involved in the rescue of eroded telomeres. Thus, the *N. castellii* ALT mechanism shows similar genetic requirements as the *S. cerevisiae* type I survivors. However, a very important distinction is the high efficiency of the mechanism in *N. castellii*, allowing the rapid reestablishment of telomere maintenance and generating a stable short telomere structure by copying the telomere-adjacent subtelomeric region to all chromosome ends.

## Materials and methods

### Strains and cell culture

*N. castellii* was previously called *Saccharomyces castellii* or *Naumovia castellii*. For this study, we developed the new *N. castellii* strain YMC700 containing deletions of the *HO* and *URA3* genes, respectively (Supplementary Fig. 1). This strain was used to create the mutant strains used in this study (Table 1; Supplementary Table 1). A pedigree was assembled to show the strain development (Supplementary Fig. 2). Strains were grown at 25 °C in YPD medium containing 1% (w/v) yeast extract, 2% (w/v) peptone, and 2% (w/v) glucose or plates additionally containing 2% (w/v) agar unless stated otherwise. Geneticin-resistant cells were screened in YPD medium containing 75-mg/L geneticin. Ura- dropout plates contained 26.7-g/L minimal SD base, 0.77-g/L ura^−^ DO supplement, and 2% agar. 5-Fluoro-orotic acid (5-FOA) plates contained 1-g/L 5-FOA, 12-µg/mL uracil, 13.35-g/L minimal SD base, and 0.385-g/L ura^−^ DO supplement.

**Table 1.** *N. castellii* key strains created and used in this study.

Strain	Ploidy	Genotype
YMC700	Diploid	*hoΔ::hphMX4, ura3Δ*
YMC701	Diploid	*hoΔ::hphMX4, ura3Δ, EST2/est2Δ*
YMC702	Haploid	*MATα, hoΔ::hphMX4, ura3Δ*
YMC704	Haploid	*MATα, hoΔ::hphMX4, ura3Δ, est2Δ*
YMC709	Diploid	*hoΔ::hphMX4, ura3Δ, TLC1/tlc1::kanMX3*
YMC730	Diploid	*hoΔ::hphMX4, ura3Δ, EST2/est2Δ, RAD52/rad52Δ::klURA3*
YMC731-733	Haploid	*hoΔ::hphMX4, ura3Δ, est2Δ, rad52Δ::klURA3*
YMC735	Diploid	*hoΔ::hphMX4, ura3Δ, EST2/est2Δ, RAD51/rad51Δ::klURA3*
YMC736-738	Haploid	*hoΔ::hphMX4, ura3Δ, est2Δ, rad51Δ::klURA3*
YMC740	Diploid	*hoΔ::hphMX4, ura3Δ, EST2/est2Δ, RAD59/rad59Δ::klURA3*
YMC741-743	Haploid	*hoΔ::hphMX4, ura3Δ, est2Δ, rad59Δ::klURA3*
YMC745	Diploid	*hoΔ::hphMX4, ura3Δ, EST2/est2Δ, RAD50/rad50Δ::klURA3*
YMC746-748	Haploid	*hoΔ::hphMX4, ura3Δ, est2Δ, rad50Δ::klURA3*
YMC750	Diploid	*hoΔ::hphMX4, ura3Δ, TLC1/tlc1::kanMX3, RAD52/rad52Δ::klURA3*
YMC751-753	Haploid	*hoΔ::hphMX4, ura3Δ, tlc1::kanMX3, rad52Δ::klURA3*
YMC755	Diploid	*hoΔ::hphMX4, ura3Δ, TLC1/tlc1::kanMX3, RAD51/rad51Δ::klURA3*
YMC756-758	Haploid	*hoΔ::hphMX4, ura3Δ, tlc1::kanMX3, rad51Δ::klURA3*
YMC760	Diploid	*hoΔ::hphMX4, ura3Δ, TLC1/tlc1::kanMX3, RAD59/rad59Δ::klURA3*
YMC761-763	Haploid	*hoΔ::hphMX4, ura3Δ, tlc1::kanMX3, rad59Δ::klURA3*
YMC765	Diploid	*hoΔ::hphMX4, ura3Δ, TLC1/tlc1::kanMX3, RAD50/rad50Δ::klURA3*
YMC766-768	Haploid	*hoΔ::hphMX4, ura3Δ, tlc1::kanMX3, rad50Δ::klURA3*
YMC770-772	Haploid	*hoΔ::hphMX4, ura3Δ*
YMC774-776	Haploid	*hoΔ::hphMX4, ura3Δ, est2Δ*
YMC777-779	Haploid	*hoΔ::hphMX4, ura3Δ, tlc1::kanMX3*

A full list of all strains is assembled in Supplementary Table 1.

Cell passaging was performed by streaking a single colony onto fresh YPD plates, and after 48-h incubation at 25 °C, single colonies were re-streaked onto new YPD plates. Re-streaking was repeated to obtain up to 16 subsequent passages. Each passage corresponds to 20 to 25 generations (Reid et al. 2002; Karademir Andersson et al. 2016). Pictures of the plates were taken in a BIORAD GelDoc XR+, and colonies were photographed with an Olympus SZ61 stereo microscope equipped with a CAM-E3CMOS6.3 camera using the ImageView software.

### Growth analysis in liquid media

For growth analysis, cells were exclusively grown in YPD media and incubated at 25 °C with 200 rpm shaking. All total cell counts were performed with the NucleoCounter YC-100 system (Chemometec) following the manufacturer's instructions. For 24-h growth analysis, a single colony taken from a YPD plate after 3 d of incubation was resuspended in 5-mL YPD and grown overnight. A 5-mL YPD culture was started at a cell density of 1 × 10^4^ cells/mL from the overnight culture, and after 24 h of growth, measurements of cell density were taken from the culture. This procedure was repeated in parallel for 10 colonies at a time. Student's *t*-test was conducted in R (R Core Team). For long-term growth analysis, cultures were started from a 100-µL YPD cell suspension of a passage 2 colony. After 24 h of growth, the cell density was measured, and the culture was diluted in 5-mL YPD to a cell density of 1 × 10^4^ cells/mL. The latter cultures were incubated as stated, and the procedure was repeated for at least 4 d for 3 individual colonies.

### Targeted gene replacement

Targeted gene replacement was performed by transformation of yeast cells with PCR-amplified DNA constructs (Supplementary Fig. 3). This transformation strategy was previously employed for the KO of the *RAD52* gene in *N. castellii* cells (Itriago et al. 2023). Deletion constructs for the *URA3*, *EST2*, *RAD52*, *RAD50*, *RAD51*, and *RAD59* genes used for transformation were designed by the authors and manufactured by GenScript. Each construct contains the klURA3 cassette flanked by the upstream and downstream sequences of the locus of the *N. castellii* gene. The klURA3 cassette contains the *K. lactis URA3* gene flanked by 143-nt-long direct repeats, allowing the subsequent pop-out of the *URA3* gene (Reid et al. 2002; Karademir Andersson et al. 2016). Cells that had recombined the klURA3 cassette out of the genome were screened in 5-FOA plates. We disrupted the *TLC1* sequence by targeted insertion of the kanMX3 cassette as previously described (Astromskas and Cohn 2009). Deletion constructs were PCR amplified using Phusion polymerase (Thermo Scientific) according to the manufacturer's instructions in 25 cycles. Primer pairs, annealing temperatures, and target sequences are listed in Supplementary Table 2. The amplification products were ethanol precipitated, resuspended in 10-mM Tris–HCl, pH 8, and quantified using a NanoDrop spectrophotometer.

Cells were transformed with the deletion construct using the lithium acetate transformation method, as previously described (Astromskas and Cohn 2009; Karademir Andersson et al. 2016). Briefly, a 50-mL YPD culture was inoculated at 3 × 10^6^ cells/mL from an overnight culture grown of a single colony. The culture was grown at 25 °C and 200 rpm shaking, until a cell density of 1 × 10^7^ cells/mL was reached. The cells were harvested by centrifugation at 2500 × *g* for 5 min, washed with sterile water, and resuspended in 25-mL transformation buffer I (0.1-M lithium acetate, 10-mM Tris–HCI, 1-mM EDTA, pH 7.5). Cells were pelleted and resuspended in 250-μL transformation buffer I. One microgram of the DNA construct was mixed with 20-µg freshly denatured salmon sperm DNA in a total volume of 10 μL and mixed with 50-μL competent cells and 300-μL transformation buffer II (40% PEG 3350, 0.1-M lithium acetate, 10-mM Tris–HCI, 1-mM EDTA, pH 7.5). The transformation mix was incubated at 25 °C for 30 min and then heat shocked at 42 °C for 10 min. After the heat shock, the cells were washed twice with sterile water and resuspended in 1-mL YPD. After an hour of incubation at 25 °C, the cells were spread on geneticin plates (for *TLC1* KO transformation) or ura^−^ dropout accordingly and incubated at 25 °C for 2 to 3 d.

### Genomic DNA extraction

The yeast genomic DNA extraction protocol from Promega genomic DNA extraction kit (Wizard Genomic DNA purification kit, REFA1125) was followed with the following modifications: 7.5 μL of 10-mg/mL Zymolyase 20T (USBiological, Z1000) were used to digest the yeast cell wall by incubating at 37 °C for 40 min, and at the final step, the DNA was diluted in 50-mM Tris–HCl (pH 8.0). DNA for each sample was quantified with Qubit 3.0 (Invitrogen), and its integrity was evaluated by resolving at least 50 ng in a 0.8% agarose gel in 0.5× TBE buffer (45-mM Tris-borate, 1-mM EDTA).

For colony PCR, DNA was extracted from cells collected by touching the surface of a single colony with a sterile blunt loop. The cells were then resuspended in 18 μL of sterile water containing a small amount of glass beads. To digest the cell wall, 2 μL of 50-mg/mL Zymolyase 20T was added to the cell suspension, followed by incubation at 37 °C for 10 min. The spheroplasts were physically disrupted by shaking the suspension containing glass beads in a Vortex-Genie (Scientific Industries) set at stage 1 to 2 for 45 to 60 s. DNA was denatured by incubating at 95 °C for 10 min followed by fast cooling on ice for 1 min. After 1-min centrifugation at 12,100 × *g*, the supernatant containing the DNA was immediately used for PCR amplification.

### Genotype analysis by PCR amplification

PCR amplifications were performed using the Phusion polymerase reaction with the primers and conditions described in Supplementary Table 2 and following the manufacturer's instructions. For colony PCR, 2 μL of the supernatant containing DNA was used as the template for the reaction. To confirm the correct insertion of the klURA3 cassette or the kanMX3 cassette after transformation, additional amplification reactions were performed utilizing internal primers targeting the sequence of the marker genes together with the corresponding flanking primers (Supplementary Table 2). PCR products were resolved in 0.6% agarose gels in 0.5× TBE.

### Column streak senescence assay

Sporulation of the diploid strains was performed as described previously (Karademir Andersson et al. 2016). Briefly, cells from an overnight culture were pelleted at 1200 × *g* for 5 min and washed twice in 2-mL sterile water. The cells were resuspended in 2-mL sporulation medium (1% potassium acetate, 0.1% yeast extract, 0.05% glucose) supplemented with 20-µg/mL uracil and incubated on a rotator at 25 °C at least 2 weeks. The production of asci was checked in a light microscope. For tetrad microdissection, 300-μL sporulation culture was centrifuged at 6700 × *g* for 2 min and washed twice with sterile water. The cells were resuspended in 60-mM phosphate buffer, pH 7.5, containing 25-mg/mL Zymolyase 20T and incubated at 37 °C for 5 min. The reaction was inactivated by gently adding 200-μL sterile water. Tetrads were dissected on YPD plates with a Singer SporePlay+ dissection microscope.

The colony streak senescence assay was based on the methodology published by Ghanem et al. (2019). Plates containing the dissected tetrads were incubated at 25 °C for 3 d. Tetrad colonies were collected with a sterile loop, resuspended in 50-µL 1× TNE (1-mM Tris–HCl, 10-mM NaCl, 0.1-mM EDTA, pH 7.4) and 15% glycerol, and placed at −80 °C after reaching a size between 0.85 and 1.00 mm in diameter. The size of the colony was determined with an Olympus SZ61 stereo microscope equipped with a CAM-E3CMOS6.3 camera using the ImageView software. Cells from the colonies were used to genotype the tetrad by colony PCR. For passaging, the cells were streaked on YPD plates directly from the tetrad colony suspension and grown for 3 d at 25 °C. Strains were further passaged using the 8-column streak plating technique, where a grid containing 8 rectangular columns of 12.5 × 25 mm is placed underneath a YPD plate and individual colonies are spread inside each column. Specifically, a colony of 0.80 to 0.85 mm in diameter was collected with a sterile loop and spread in the column area from the top down in a zig-zag motion, once at the bottom of the column the loop was lifted, and the spreading was repeated 3 times always using the same face of the loop. The cells were then spread in a zig-zag motion from the bottom up 1 time. Lastly, the cells were spread 3 times from the top down as before. Cells were then incubated at 25 °C for 3 d and the plates photographed with a BIO-RAD GelDoc XR+. The image was exported as a TIF file using the BIO-RAD Image Lab software. A total of 48 colonies were spread in 6 plates for 3 haploid strains obtained from the tetrad dissection (16 colonies or 2 plates per strain). To evaluate and quantify the growth of each colony in the column area, first the shape of the column was imposed on the full-size picture using Adobe Illustrator, and the area was slightly reduced (80% of the size) to account for errors while plating. The growth of the cells in the column was then scored by visual evaluation by 8 trained persons as having full growth if the lawn of cells had confluent growth throughout the area of the column, meaning that it had a consistent growth pattern without internal gaps or lack of growth toward the ends. Full growth scoring was awarded even if the lawn of cells was not extremely dense, if it followed the established criteria. Quantifications were made by calculating the average number of full columns per plate for each genotype. The evaluation of the scoring strictly followed the criteria, resulting in the wild-type (WT) strains not obtaining a maximum 8.0, due to variabilities in growth and handling in the technical repeats. The data collected in Microsoft Excel was analyzed by Student's *t*-test in R.

### TRF assay

A total of 300 ng of genomic DNA was digested by *Hinf*I (Thermo Scientific) at 37 °C for 1.5 h. Digested DNA was resolved by agarose gel electrophoresis with a constant voltage of 1.5 V/cm for 16 h at 4 °C in a 0.8% gel casted in 0.5× TBE. The DNA was transferred to a positively charged nylon membrane (Hybond-XL, Amersham) by Southern blot using the Trans-blot SD Semi-Dry Transfer Cell (Bio-Rad) system. The blotted membrane was rinsed with 2× SSC, air-dried, and crosslinked by exposing it to 120 mJ/cm^2^ of UV light. Hybridization probes were 5′ end labeled by T4 polynucleotide kinase and [γ32P]-ATP. The membrane was hybridized overnight at 40 °C with a telomeric probe 5′-(TGTCTGGG)_2_-3′ or a subtelomeric probe 5′-GTTGGAGGTATTTGTTGTTGATG-3′ targeting the TelKO element in hybridization solution (250-mM Na_2_HPO_4_, 7% SDS, 1-mM EDTA). The membranes were washed twice for 20 min at 40 °C with washing solution (100 mM Na_2_HPO_4_, 2% SDS). Membranes were then exposed to phosphorimager screens and scanned using a Typhoon FLA 9500 biomolecular imager (Cytiva). To remove the probe, the membranes were washed 2 times with boiling 0.1% SDS and then washed 2 times with 2× SSC. Displayed images were converted from raw data Gel file to TIFF file using the ImageQuant software, contrast adjusted in ImageJ and the figures assembled in Adobe Illustrator.

## Results

### *N. castellii* telomerase KO cells rapidly activate the ALT mechanism

**Fig. 1.**
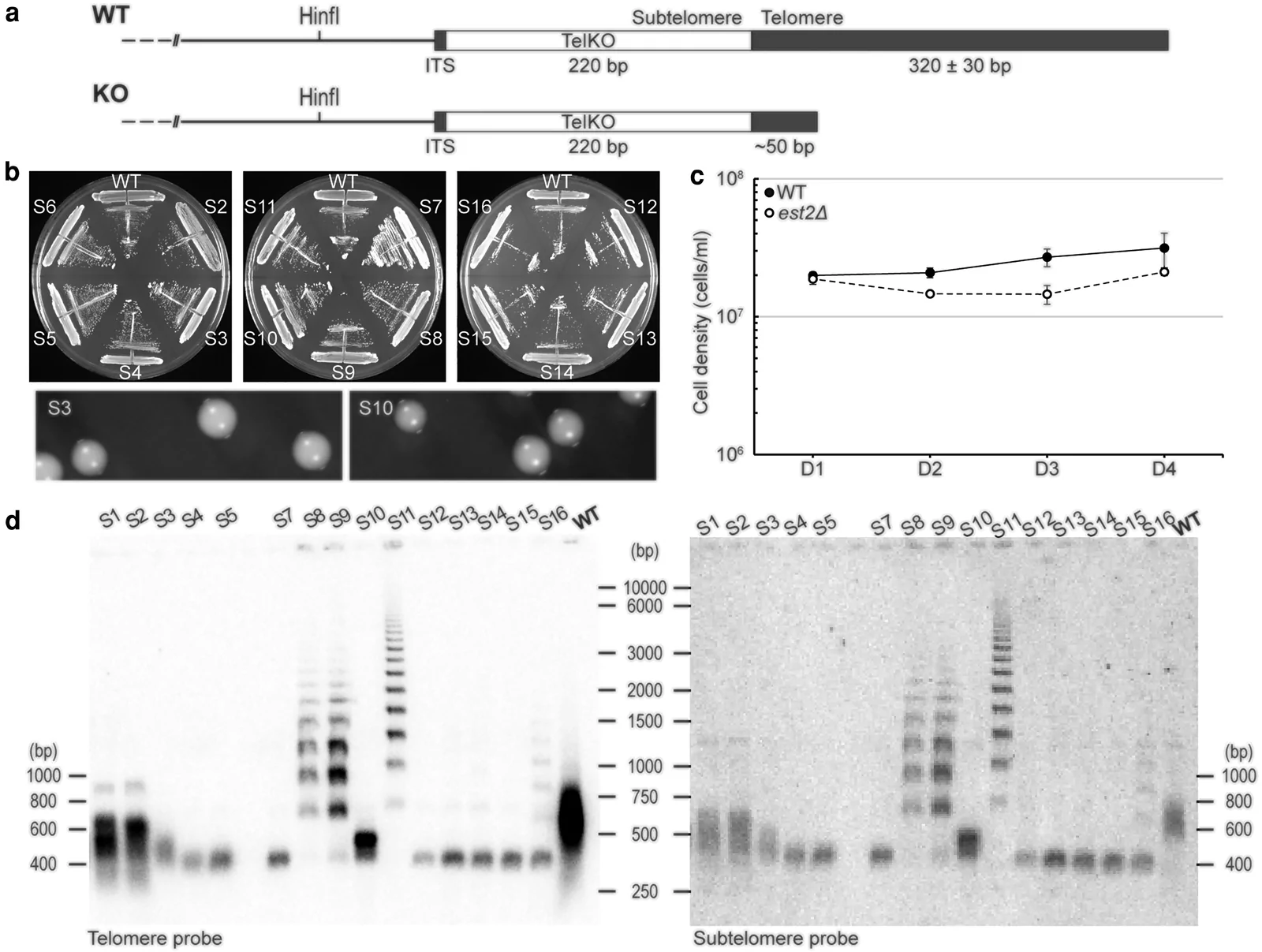
*N. castellii est2Δ* cells rapidly activate the ALT mechanism and maintain short telomeres. a) Schematic representation of the telomeres of WT and telomerase-deficient (KO) strains. The subtelomeric TelKO element is located directly adjacent to the telomeric tract in several chromosome ends. In the WT strain, the telomeric double-stranded region is 320 ± 20 bp, and each end harbors a single TelKO copy, with TelKO variants of different lengths residing in separate chromosome ends. The TelKO element is flanked by a short 9-bp ITS and a cleavage site for the enzyme *Hinf*I. The sequences internally of ITS have not been studied in detail, but show some extent of similarity between different subtelomeres. In KO strains, the telomeres shorten rapidly, and a specific TelKO element is copied and spread to the other telomeres. Thus, the ALT mechanism generates homogeneous chromosome ends that contain the TelKO sequence followed by a short ∼50-bp telomeric stretch. b) Upper: Growth analysis of the haploid *est2Δ* strain YMC704. The KO strain and the isogenic WT strain from the same tetrad were continuously streaked onto YPD plates for ∼320 generations (S1 to S16). Lower: Close-up pictures of representative *est2Δ* colonies from S3 and S10. c) Continuous growth analysis of the *est2Δ* strain YMC704 and the isogenic WT strain in liquid YPD medium. Cultures were inoculated with cells from S2 to a starting density of 1 × 10^4^ cells/mL. Cell density was measured after 24 h of incubation, and the cultures were then diluted to the initial density and re-incubated. The procedure was repeated for 4 d (D1 to D4), and the graph shows the average values for WT (filled circle, solid line) and *est2Δ* (empty circle, dotted line). Error bars represent the SEM (*n* = 3). d) TRF assay of the *est2Δ* strain YMC704. Genomic DNA from S1 to S16 was digested with *Hinf*I, resolved in a 0.8% agarose gel and analyzed by Southern blot hybridization with a 16-mer telomeric sequence probe (left). The membrane was then stripped and re-hybridized with a 23-mer subtelomeric probe (right) targeting the TelKO sequence.

In the absence of telomerase activity, some cells are capable of activating ALT mechanisms to maintain a functional telomere structure, thus preventing the lethal consequences of genome instability associated with telomere shortening. The budding yeast *N. castellii* effectively activates ALT and bypasses senescence when the *TLC1* gene is disrupted, a process involving the spreading of the subtelomeric TelKO element to all chromosome ends (Fig. 1a) (Cohn et al. 2019; Jaiswal et al. 2025). Thus, the ALT mechanism generates homogeneous chromosome ends that contain the TelKO sequence followed by a short telomeric stretch (Fig. 1a). Here, we developed a new line of *N. castellii* ALT strains by deletion of the *EST2* gene, encoding for the reverse transcriptase component of telomerase. We utilized targeted gene replacement by creating a DNA construct containing the *K. lactis URA3* cassette (klURA3) flanked by the upstream and downstream sequences of the *N. castellii EST2* gene (Supplementary Fig. 3). We selected our recently developed diploid strain YMC700 (*hoΔ::hphMX4, ura3Δ*) as the parental strain for this study and successfully deleted a single copy of the *EST2* gene. Several heterozygous colonies were plated on 5-FOA to select for cells that lost the marker gene through recombination of direct repeats present in the klURA3 cassette. The resulting heterozygous strain YMC701 (*hoΔ::hphMX4, ura3Δ, EST2/est2Δ*) maintains WT telomeres, indicating that a single copy of *EST2* is sufficient for the maintenance of telomeres by telomerase (Supplementary Fig. 4).

To obtain haploid *est2Δ* telomerase-deficient strains, we sporulated the heterozygous strain YMC701 and dissected the spore tetrads. Germination of the spores and colony growth resulted in the first streak, S1. To evaluate the *est2Δ* phenotype, colonies were re-streaked on YPD plates every 2 d. Three different strains were passaged for a total of 16 streaks (S1 to S16), corresponding to >320 generations of growth. Consistent with our previous observations of telomerase-deficient strains (Cohn et al. 2019), we did not detect any severe growth defect during the passaging of the *est2Δ* strains, nor any significant difference in colony morphology compared to isogenic WT haploid strains (Fig. 1b). To further evaluate the growth capabilities of *est2Δ* mutants, we performed a continuous growth analysis in liquid media. We started the cultures at a cell density of 1 × 10^4^ cells/mL from streak 2 (S2) colonies and measured the cell density after 24 h of growth, before diluting the cultures and repeating the process for a total of 4 d. The results showed that *est2Δ* cells have only slightly lower growth rate compared to isogenic WT strains (Fig. 1c). Notably, in contrast to *S. cerevisiae* telomerase-deficient strains, we did not observe any severe decrease in growth rate. Instead, *N. castellii est2Δ* cells maintain a growth ability that is only slightly reduced also during continuous passaging in liquid media (Fig. 1c).

To study the telomere length in our new *est2Δ* strains, we performed TRF assays on colonies from each streak of the serial plate re-streaking procedure. Genomic DNA was extracted from a single colony of each of the different streaks (S1 to S16) and digested with the *Hinf*I restriction enzyme. The DNA fragments were resolved in an agarose gel and transferred to a nylon membrane. Telomeric fragments were visualized by hybridization with a 16-mer telomeric sequence probe and radioactive imaging (Fig. 1d, left). The membrane was then stripped and re-hybridized with a subtelomeric probe targeting the TelKO element (Fig. 1d, right). The *Hinf*I enzyme has a cutting site near the end of the subtelomeric region, allowing for the accumulation of telomeric fragments in a single smeared band with a mean length of ∼600 bp (Fig. 1). Such smeared bands are characteristic of telomeric fragments, representing the length variation between individual telomeres in the cell and the variation within the population of cells. When comparing to the telomere length of the WT strain, we observed a drastic shortening of the *est2Δ* cell telomeres as early as S1 to S2, corresponding to 20 to 40 generations after the deletion of the *EST2* gene (Fig. 1d, left). Generally, after these passages, the telomeres are stabilized, and telomere fragments accumulate with a length of ∼400 bp (Fig. 1d; Supplementary Fig. 5). The narrow and distinct short terminal fragments found after the loss of telomerase activity contain subtelomeric sequences (Fig. 1d, right). The results indicate that after 40 generations, short telomeres are being maintained by the ALT mechanism, which is in line with our previous observations in telomerase-deficient strains (Cohn et al. 2019; Jaiswal et al. 2025).

The presence of the ALT mechanism in our *est2Δ* mutants is further confirmed by the appearance of the dramatic change in banding pattern visible for some of the streaks (Fig. 1d; Supplementary Figs. 5 and 6). This banding pattern, termed a “ladder pattern” due to the stepwise increase in the length of the bands, is characteristic of the ALT mechanism of *N. castellii* and represents the consecutive addition of repeats containing the TelKO element onto the telomeres (Cohn et al. 2019; Jaiswal et al. 2025). The appearance of the ladder pattern is a stochastic event representing the mechanism in which ALT cells process telomeres. In Fig. 1d, the ladder patterns observed have ∼300-bp increments in each step, which is consistent with previous observations (Cohn et al. 2019; Jaiswal et al. 2025). We previously showed that this corresponds to the addition of composite repeats composed of the TelKO element and telomeric sequence stretches, resulting in long arrays of tandem repeats (Jaiswal et al. 2025). To further confirm that the ALT mechanism in our new strains corresponds to the previously observed, we developed telomerase-deficient mutants by disrupting the *TLC1* gene in our newest strain line using the geneticin resistance gene cassette, as demonstrated previously (Cohn et al. 2019). In the colony streak assays and TRF assays, these *tlc1^−^* strains exhibit similar features, where telomeres quickly shorten and later accumulate in a ∼400-bp band that is maintained for over 320 generations (Supplementary Fig. 6). Moreover, the characteristic ladder pattern appeared in 1 of the strains (Supplementary Fig. 6).

In summary, we developed telomerase-deficient strains of the budding yeast *N. castellii* by targeted gene replacement transformation of the *EST2* gene and KO disruption of the *TLC1* gene, coding for the respective core components of telomerase. Our results show that telomerase-deficient *N. castellii* strains do not exhibit any severe growth phenotype when passaged for an extensive number of generations. Furthermore, analysis of the telomere structure reveals that the cells undergo the rapid changes in telomere structure and maintenance that are characteristic of the activation of the ALT mechanism in *N. castellii* (Cohn et al. 2019; Jaiswal et al. 2025).

### The *N. castellii* ALT cells are dependent on the *RAD52* gene

**Fig. 2.**
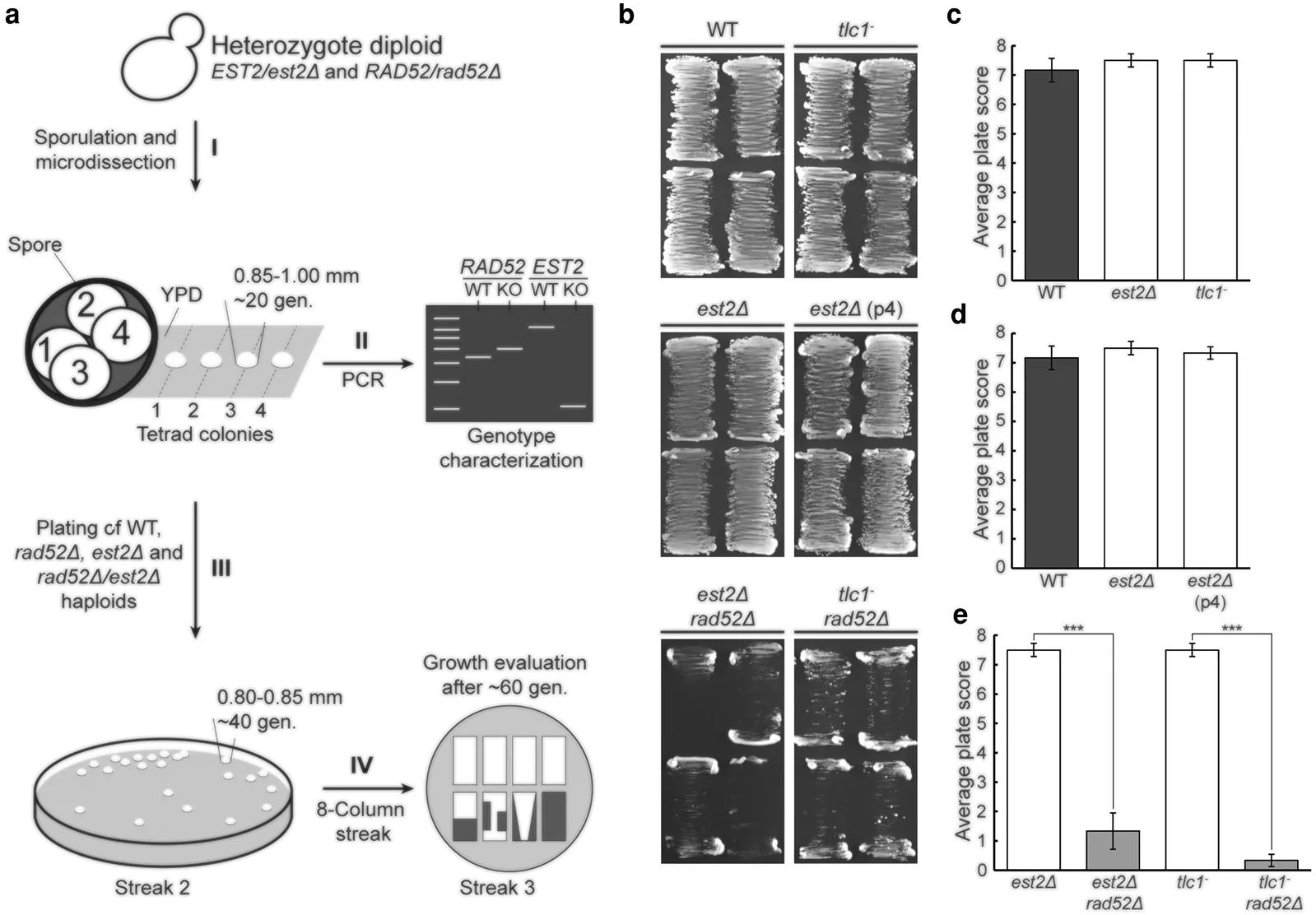
*RAD52* is necessary for the sustained growth of *N. castellii* telomerase-deficient strains. a) Schematic of the column streak senescence assay for quantitative growth assessment. Diploid heterozygous strains containing a functional and KO copy of the genes of interest, in this example *EST2* and *RAD52*, were first sporulated and the spore tetrads dissected to generate mutant haploid strains (I). Dissected tetrads were placed on a YPD plate and incubated for 3 d at 25 °C to allow for colony formation. Colonies were collected and stocked when reaching a size of 0.80 to 1.00 mm in diameter, after ∼20 generations of growth. The genotype was screened by colony PCR (II). Double heterozygous strains can produce 4 different genotypes, in our example: *EST2/RAD52* (WT), *EST2/rad52Δ* (*rad52Δ*), *est2Δ/RAD52* (*est2Δ*), and *est2Δ/rad52Δ* (double-KO). PCR genotyping was performed with primers flanking the gene locus of interest, giving different lengths of the WT and KO bands of PCR amplicons in agarose gel electrophoresis. Strains from the different genotypes were streaked on YPD plates and incubated for 3 d at 25 °C (III), to reach a size of 0.80 to 0.85 mm in diameter, corresponding to ∼40 generations after the germination of the spores. Growth capacity was evaluated by the column streak senescence assay (IV), where individual colonies were evenly spread in a single column, in a grid containing 8 columns per plate (48 colonies per genotype). After incubation for 3 d at 25 °C (creating S3, in total ∼60 generations), growth ability was scored as the number of columns showing confluent growth (white area denotes growth in schematic plate). b) Column streak senescence assay results for telomerase-deficient and double-KO haploids. The diploid strains YMC730 and YMC750 were sporulated, and for each genotype, we picked 3 different strains for the S2 streaks on YPD plates. For each strain, 16 columns were plated in 2 plates of the 8-column streak (3 biological samples, 48 columns per genotype in total). c to e) Quantitative analysis of the column streak senescence assay: (c) telomerase-deficient strains, (d) S4 colonies of the *est2Δ* strain; S2 colonies were re-streaked and then 48 such S3 colonies were used for the 8-column streak assay. (e) Double-KO strains with *RAD52*. The diagrams show the average number of full columns per plate for each genotype. Error bars represent SEM for all plates (*n* = 6). Difference in the means was calculated using the 2-sided Student's *t*-test; significance is represented by “***” when *P* < 0.001.

After losing telomerase activity, *S. cerevisiae* cells can survive and escape replicative senescence by activating ALT mechanisms, which depend on Rad52-mediated homologous recombination, to reestablish telomere maintenance by addition of subtelomeric or telomeric repeats (Lundblad and Blackburn 1993). Here, we aimed to investigate if the establishment of the ALT mechanism in *N. castellii* requires homologous recombination mediated by Rad52. The *N. castellii RAD52* gene was previously identified and characterized (Itriago et al. 2023). Utilizing the same targeted deletion transformation strategy as described earlier, we knocked out the *RAD52* gene in the YMC701 strain, generating a double heterozygous strain for the *EST2* and *RAD52* genes, respectively (YMC730). We confirmed by TRF assay that the telomere structure of YMC730 remained unaltered (Supplementary Fig. 7). We then sporulated YMC730 and dissected the spore tetrads on YPD plates (Fig. 2a.I). Once germinated, the tetrad colonies were collected and stocked after reaching a size between 0.85 and 1.00 mm in diameter. The colony size was measured to standardize the number of generations that the cells have gone through, from a single cell to a full-rounded medium sized colony, which we estimate to be ∼20 generations based on the generation time of *N. castellii*. We genotyped the tetrad colonies by colony PCR targeting the locus of the *EST2* and *RAD52* genes, respectively (Fig. 2a.II). As expected, 4 different types of haploid strains were identified, corresponding to the *EST2/RAD52* (WT), *EST2/rad52Δ* (*rad52Δ*), *est2Δ/RAD52* (*est2Δ*), and *rad52Δ/est2Δ* (double-KO) genotypes. Cells from the stock of each genotype were streaked on YPD plates and grown for 3 d at 25 °C (Fig. 2a.III). In parallel, we created a *TLC1* heterozygous strain (YMC709) by targeted disruption of the YMC700 strain with the geneticin resistance gene cassette. We then knocked out the *RAD52* gene in YMC709 to generate a double heterozygous strain for *TLC1* and *RAD52* genes (YMC750) and followed the same procedure to obtain additional complementary data.

From the moment of germination, the S2 cells have grown for ∼40 generations (Fig. 2a.III). To quantitatively assess the growth ability of each mutant strain, we performed a column streak senescence assay (Ghanem et al. 2019). Here, we plated colonies of 0.80 to 0.85-mm diameter in size individually on a YPD plate within a grid pattern of 8 columns (Fig. 2a.IV). The colonies were spread evenly across the area of the column (described in the Materials and methods section). Forty-eight individual colonies for each strain were plated in the column streak senescence assay, to account for strain variability in the appearance of the phenotypes (Ghanem et al. 2019). After 3 d of incubation at 25 °C, the growth of the cells was evaluated by the ability of each individual colony to fill either the whole column area or only part of it (top and bottom columns of schematic plate in Fig. 2a, respectively). The growth was assessed individually for all strains (Fig. 2b), and the quantification and graphical presentation was made by utilizing the plate containing 8 columns as the unit of measurement (Fig. 2c to e).

First, after ∼60 generations of growth (S3), both *est2Δ* and *tlc1^−^* strains are able to grow and fill the area of the column comparably equal to the isogenic WT strain (Fig. 2b). Indeed, they show a consistent result in the quantification (Fig. 2c). These results are in accordance with what we have previously observed in our colony serial streaking assays. In addition, we performed the column streak senescence assay for *est2Δ* colonies from S4 (*est2Δ* S4) and found no difference in growth compared to the isogenic WT strain or the *est2Δ* S3 colonies (Fig. 2b and d). Furthermore, we found no difference in growth between the *rad52Δ* strains and the WT isogenic strain, indicating that the loss of *RAD52* did not affect the growth capacity under these conditions (Supplementary Table 3).

Notably, our results show that most telomerase-deficient double-KO colonies were unable to sustain growth at the third streak (S3), indicating that the cells cannot proliferate beyond ∼60 generations on solid media (Fig. 2b and e). Out of the 48 colonies plated, only 8 colonies of the *est2Δ/rad52Δ* and 2 colonies of the *tlc1^−^/rad52Δ* strains covered the full area of the column. The reduced growth capability is evident from the quantitative analysis (Fig. 2e). Here, we found a very significant difference in the growth of the double-KO strains when compared to the single-KO telomerase-deficient isogenic strains. Based on the results, double-KO strains drastically lose their growth abilities after sequential passaging, and therefore *RAD52* gene function is essential for telomerase-deficient strains to sustain long-term growth.

In summary, here we created *N. castellii rad52Δ* strains and combined them with the telomerase-deficient strains to obtain double-KO strains (*est2Δ/rad52Δ*, *tlc1^−^/rad52Δ*). By quantitative growth assessment using the column streak assay, we show that the ability of *N. castellii* telomerase-deficient strains to sustain long-term growth without a growth crisis is clearly dependent on Rad52, hence implicating that the *N. castellii* ALT mechanism is mediated by homologous recombination.

### *N. castellii* ALT cells utilize a *RAD51*-dependent mechanism to achieve long-term growth

A genetic requirement of *RAD52* for the survival of telomerase-deficient cells indicates that the establishment of the *N. castellii* ALT is mediated by a homologous recombination mechanism. Break-induced replication (BIR) is a noncanonical and error-prone homology-directed repair mechanism that is very common at telomeres, while it rarely occurs to repair DNA damage in the rest of the genome. Indeed, *S. cerevisiae* ALT mechanisms utilize 1 of 2 types of *RAD52*-dependent BIR mechanisms to maintain the telomeres (Chen et al. 2001). In the *RAD51*-dependent BIR mechanism (type I), Y’ elements from the subtelomeric region are copied over the telomeres by homologous recombination involving large segments of homology. In the *RAD51*-independent BIR mechanism (type II), recombination occurs between the TG_1–3_ repeats of telomeres through short-homology recombination mediated by Rad59 and Rad50.

**Fig. 3.**
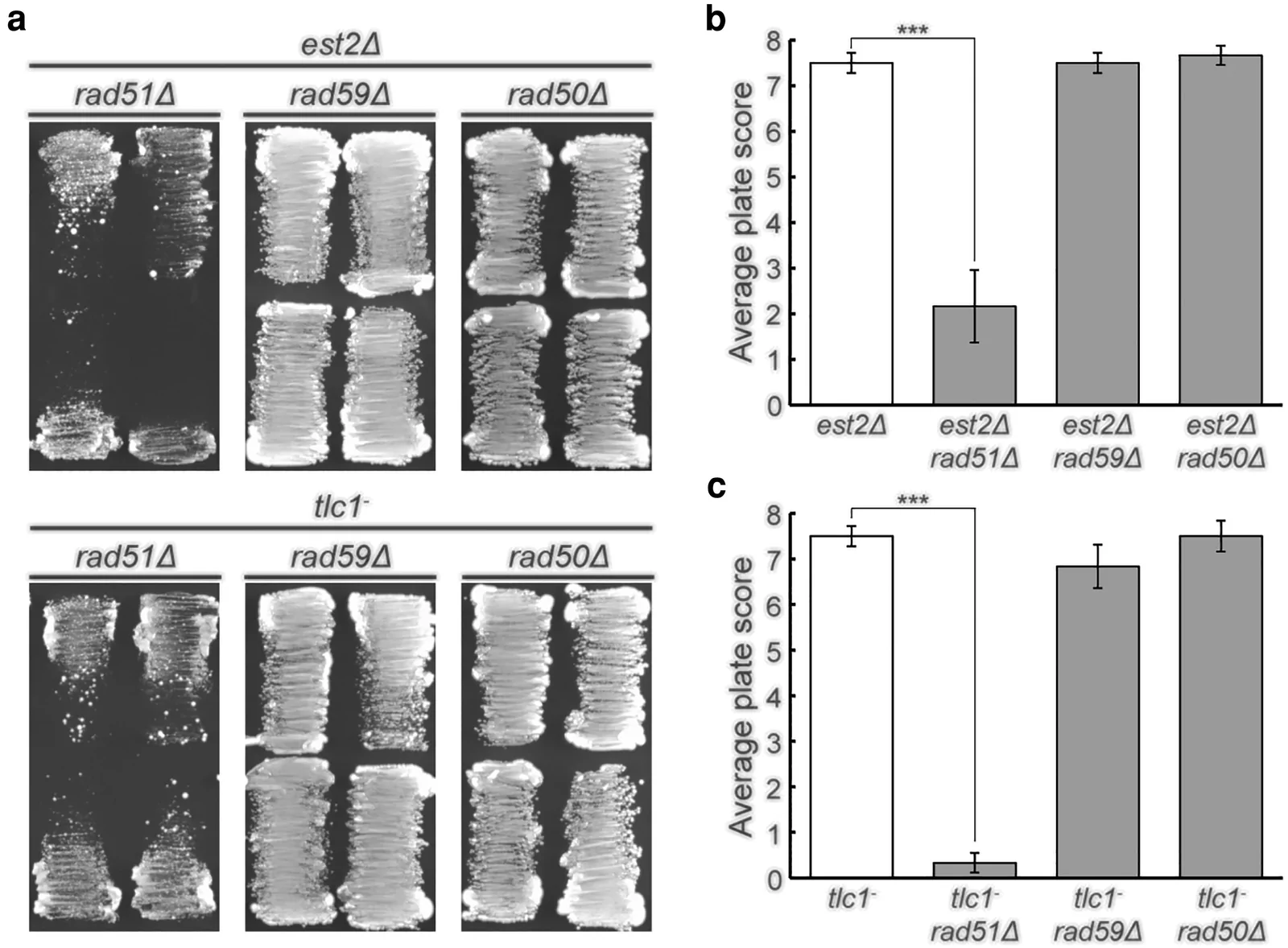
*RAD51*, but not *RAD59* or *RAD50*, is necessary for the establishment of ALT in *N. castellii*. a) Column streak senescence assay for telomerase-deficient and double-KO haploid strains, starting with the sporulation and tetrad dissection of the diploid strains YMC735, YMC740, YMC745, YMC755, YMC760, and YMC765. Per double-KO genotype, we selected 3 different strains to be used for S2 streaks. For each strain, 16 columns were plated (3 biological samples, total of 48 columns per genotype). b and c) Quantitative analysis of the column streak senescence assay for double-KO haploids of *RAD51*, *RAD51*, or *RAD59* as indicated, in backgrounds of *est2Δ* (b) vs *tlc1^−^* (c). The diagrams show the average number of columns with full growth per plate for the respective genotype. Error bars represent SEM for all plates (*n* = 6). Difference in the means was calculated using the 2-sided Student's *t*-test; significance is represented by “***” when *P* < 0.001.

To investigate if *N. castellii* ALT cells utilize 1 of these mechanisms of BIR to maintain the telomeres, we first identified the homologs of the respective *RAD50*, *RAD51*, and *RAD59* genes in *N. castellii* by their high sequence identity to the respective *S. cerevisiae* genes (Supplementary Fig. 8). Next, by gene replacement transformation of the YMC701 strain, we created KO strains of the respective genes, as well as double heterozygous strains for the *EST2* and *RAD51*, *RAD59*, and *RAD50* genes, respectively (YMC735, YMC740, and YMC745). Similarly, transformation of the YMC709 strain was carried out to generate diploid heterozygous strains for *TLC1* and the *RAD51*, *RAD59*, and *RAD50* genes, respectively (YMC755, YMC760, and YMC765). We checked the telomere structure of the diploid heterozygous strains and found no difference compared to the WT (Supplementary Fig. 7). All double heterozygous strains were sporulated and the spore tetrads dissected, collected, and characterized by PCR using primers targeting the locus of the corresponding genes (Supplementary Table 2). After characterization of the tetrad colonies, cells with the respective double-KO genotypes were grown in the column streak assay described above (*est2Δ/rad51Δ*, *est2Δ/rad59Δ*, *est2Δ/rad50Δ*, *tlc1^−^/rad51Δ*, *tlc1^−^/rad59Δ*, *tlc1^−^/rad50Δ*, *rad51Δ*, *rad59Δ*, and *rad50Δ*) (Fig. 3).

A dramatic decrease in growth was observed for the telomerase-deficient and *rad51Δ* double-KO strains, which is in stark contrast to the good growth capabilities of the double-KO strains for both the *rad59Δ* and *rad50Δ* strains (Fig. 3a). Indeed, when evaluating the growth in the columns of each plate, we observed a very significant difference in growth of the *rad51Δ* double-KO mutant strains compared to the growth of the telomerase-deficient strains (Fig. 3b and c). No significant difference in growth capability was observed for the *rad59Δ* or *rad50Δ* double-KO mutants compared to the telomerase-deficient strains (Fig. 3b and c). Moreover, no significant difference in growth was observed for the single mutant strains for each recombination gene when compared to the isogenic WT strain (Supplementary Table 3). Together, these results indicate that *RAD51* gene function is necessary to sustain long-term growth in *N. castellii* telomerase-deficient cells.

The column streak assays were quantitatively analyzed by scoring the growth capacity (confluent growth/nonconfluent growth) within the columns (Table 2). The number of colonies that successfully showed confluent growth was counted, and then the average plate score was calculated with the corresponding SD of the mean. The number of colonies that successfully filled the area of the columns in the plate was counted, as well as the average score of colonies grown in each individual plate, with the corresponding SD of the mean. The median values for each data set show that the mean values are not deviating from the general distribution of the values in each sample set. The standard deviation was high in comparison to the mean for samples that showed a drastically lower growth in the column assay (*RAD52* and *RAD51* double-KO strains), representing the variability of the stochastic nature of cell senescence, i.e. the number of cell cycles that each cell can complete before losing its ability to divide (Ghanem et al. 2019). Interestingly, *RAD52* and *RAD51* double-KO mutants of the *tlc1^−^* background show somewhat lower growth capabilities than the cells from the *est2Δ* background (Table 2).

**Table 2.** Summary of the column streak senescence assay results.

	Comparison of growth for telomerase-deficient strains			
	WT	*est2Δ*	*est2Δ* (S4)	*tlc1^−^*
Full columns	43/48	45/48	44/48	45/48
Average plate score	7.2 ± 0.4	7.5 ± 0.2	7.3 ± 0.2	7.5 ± 0.2
Median	7.5	7.5	7	7.5
Difference to WT		n.s.	n.s.	n.s.

	Comparison of growth for *EST2* double-KO mutant strains			
	*est2Δ* *rad52Δ*	*est2Δ* *rad51Δ*	*est2Δ* *rad59Δ*	*est2Δ* *rad50Δ*
Full columns	8/48	13/48	45/48	46/48
Average plate score	1.3 ± 0.6	2.2 ± 0.8	7.5 ± 0.2	7.7 ± 0.2
Median	1	2.5	7.5	8
Difference to *est2Δ*	*P* = 0.000	*P* = 0.000	n.s.	n.s.

	Comparison of growth for *TLC1* double-KO mutant strains			
	*tlc1^−^rad52Δ*	*tlc1^−^rad51Δ*	*tlc1^−^rad59Δ*	*tlc1^−^rad50Δ*
Full columns	2/48	2/48	41/45	45/48
Average plate score	0.3 ± 0.2	0.3 ± 0.2	6.8 ± 0.5	7.5 ± 0.3
Median	0	0	7	8
Difference to *tlc1^−^*	*P* = 0.000	*P* = 0.000	n.s.	n.s.

The number of columns with confluent growth is summarized for each of the strain genotypes out of the total amount of columns plated. The average plate score (average number per plate) is presented together with SEM for all plates (*n* = 6). Difference in the means was calculated using the 2-sided Student's *t*-test for the telomerase-deficient strains compared to the WT, or double-KO strains compared to the telomerase-deficient single-KO mutant as indicated.

n.s., nonsignificant differences.

In summary, we have collected clear and strong data regarding the lack of a senescence phenotype in *N. castellii* telomerase-deficient strains and the genetic requirements of its ALT mechanism. The data presented quantitatively demonstrates that telomerase-deficient strains do not experience a growth crisis and can sustain WT-like growth on solid media after 60 to 80 generations (Fig. 2; Table 2). Moreover, we have no indication that a growth crisis event occurs earlier than this time point, based on our observations of colonies after germination of the spores and of colony morphology after S2 (further discussed below). We found similar growth capabilities to the telomerase-deficient mutants in double-KO mutant strains of the *RAD59* and *RAD50* genes, suggesting that the cells can still activate the ALT mechanism and proliferate in the absence of these genes (Fig. 3; Table 2). Notably, we found that both *RAD52* and *RAD51* are necessary for the establishment of the ALT mechanism in *N. castellii*, and thus mutants that are telomerase-deficient and lack 1 of these 2 genes are unable to sustain long-term growth, showing senescence after 60 or less generations of growth (Figs. 2 and 3; Table 2). These results are supported by the importance of the *RAD51* gene in DNA damage repair (Supplementary Fig. 9). Our gene characterization showed that this function is conserved in *N. castellii*, since the *RAD51*-deficient strains exhibit an increased sensitivity to UV light irradiation and exposure to the genotoxic agent bleomycin.

### Recombination-deficient strains undergo a strong senescence phenotype

**Fig. 4.**
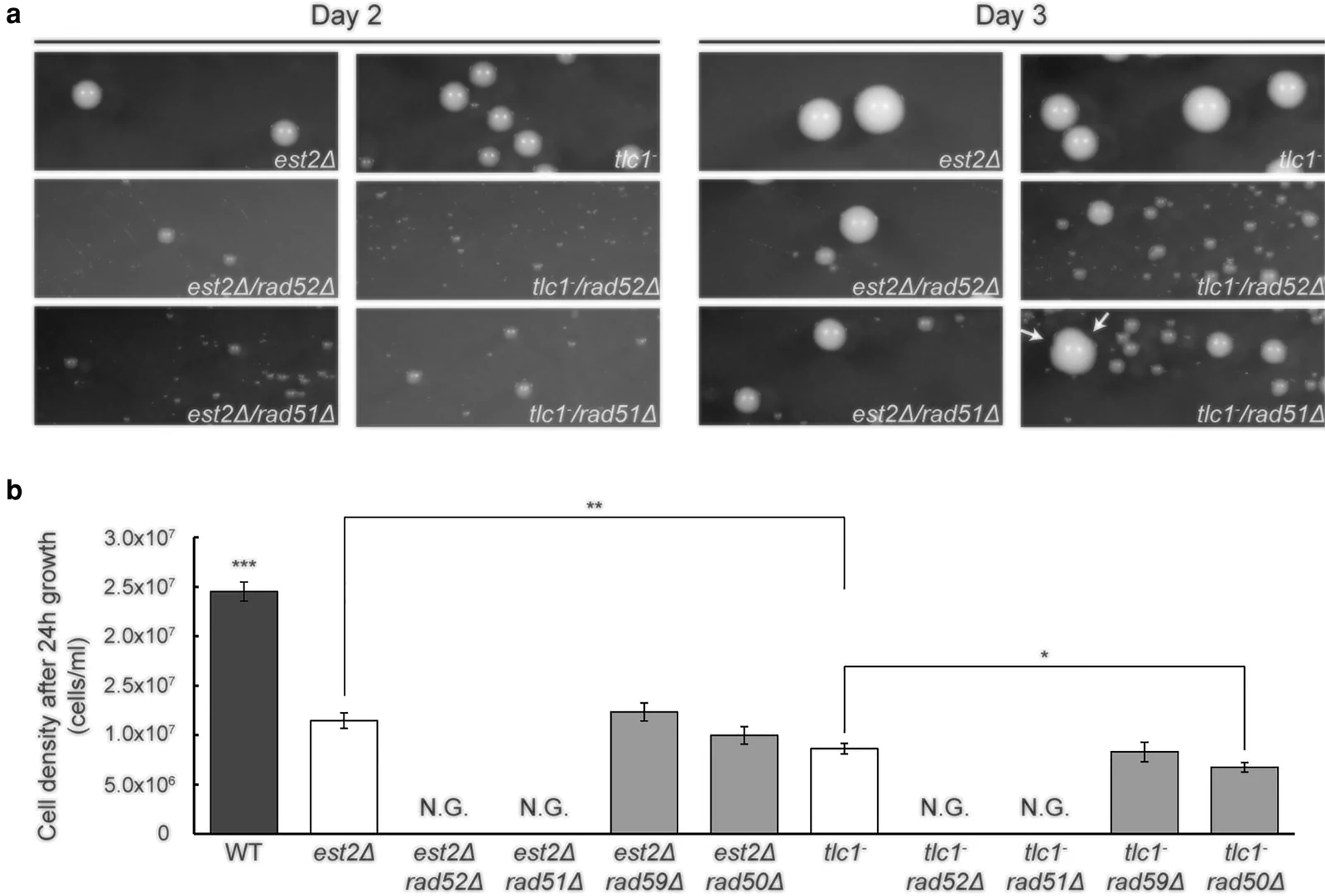
Telomerase-deficient double mutants with either *rad52Δ* or *rad51Δ* show early signs of senescence. a) Close-up photographs of double-KO mutant strains at streak 2 (S2) after 2 and 3 d of incubation. Cells from the tetrad colonies (S1) were plated on YPD media and incubated at 25 °C for the indicated times. Random representative colonies are shown for days 2 and 3, respectively. Arrows indicate 1 of the rare colonies with a clearly dented morphology. b) Twenty-four-hour growth analysis for telomerase-deficient and double-KO strains. Overnight cultures started from 10 individual S2 colonies were used to start 5-mL YPD cultures at a cell density of 1 × 10^4^ cells/mL. The bars represent the average cell density achieved by the cultures after 24 h of growth. N.G. indicates no growth. Error bars represent SEM for all samples (*n* = 10).

The marked growth deficiency observed for the double-KO mutants (telomerase-deficient combined with either *rad52Δ* or *rad51Δ*) suggests that these strains are unable to utilize the ALT mechanism. No deviating phenotype of these mutant cells was detectable during the germination of the spores, and the majority of the dissected spores reached the correct colony size after 3 d. On S2 plates, double-KO cells with either *RAD52* or *RAD51* deficiency required longer time to grow into middle-size colonies than the solely telomerase-deficient ALT strains (Fig. 4a). Indeed, while we normally passage cells after 2 d of incubation for the serial streaking assay, an additional day of incubation was added to the column streak assay to allow for the growth of colonies from these recombination-deficient double-KO strains. Even after 3 d of growth, a significant number of colonies had a size smaller than 0.80 mm in diameter, indicating that they could not achieve 40 generations of growth by this time. Some *RAD52*- and *RAD51*-deficient double-KO colonies exhibited irregular colony morphology characterized by unevenly shaped colonies and, occasionally, dents when grown to a larger size (Fig. 4a). This phenotype, while not predominant, contrasts the rounded and smooth-edged colonies of the WT and telomerase-deficient isogenic strains (Fig. 4a).

To further study the early differences in growth observed in the strains deficient for ALT, we performed 24-h growth assays of the different haploid strains. In this assay, 10 colonies picked from S2 plates were used to start the overnight YPD cultures. The starter culture was diluted to a density of 1 × 10^4^ cells/mL, and after 24 h of incubation, the cell density was measured, and the average was calculated (Fig. 4b). The average growth obtained for the WT strain corresponds to ∼11 generations of the cells in culture, giving a value significantly higher than the average growth value obtained for all other strains. Solely telomerase-deficient strains exhibit a lower overall growth rate compared to WT cells, and similar as seen before (Table 2), *tlc1^−^* mutants have slight but significant lower overall growth than the *est2Δ* mutants (Fig. 4b). It was not possible to assay the *RAD52*- and *RAD51*-deficient double-KO strains because overnight cultures did not show any growth even after prolonged incubation, consistent with our observations described above (Table 2). Moreover, there was no difference between the growth of *est2Δ* and the double-KO with *RAD50* or *RAD59*. We also did not find any difference in growth between *tlc1^−^* and *tlc1^−^ rad59Δ* strains; however, the *tlc1^−^ rad50Δ* strain grew slightly less than the *tlc1^−^* strain.

In summary, telomerase-deficient strains (*est2Δ* and *tlc1^−^*) exhibit somewhat lower growth in liquid media compared to the WT isogenic strains. However, this reduction in growth rate does not affect their ability to grow on YPD plates and form colonies with WT-like morphology. The *RAD59* and *RAD50* double-KO mutants exhibit similar growth as the solely telomerase-deficient strains. However, strains lacking telomerase activity in combination with loss of *RAD52* or *RAD51* gene function show a very early onset of senescence phenotypes, defined by poor growth on YPD plates, irregular colony shapes, and the inability to grow in liquid cultures.

### The ALT mechanism rapidly modifies the telomere structure

**Fig. 5.**
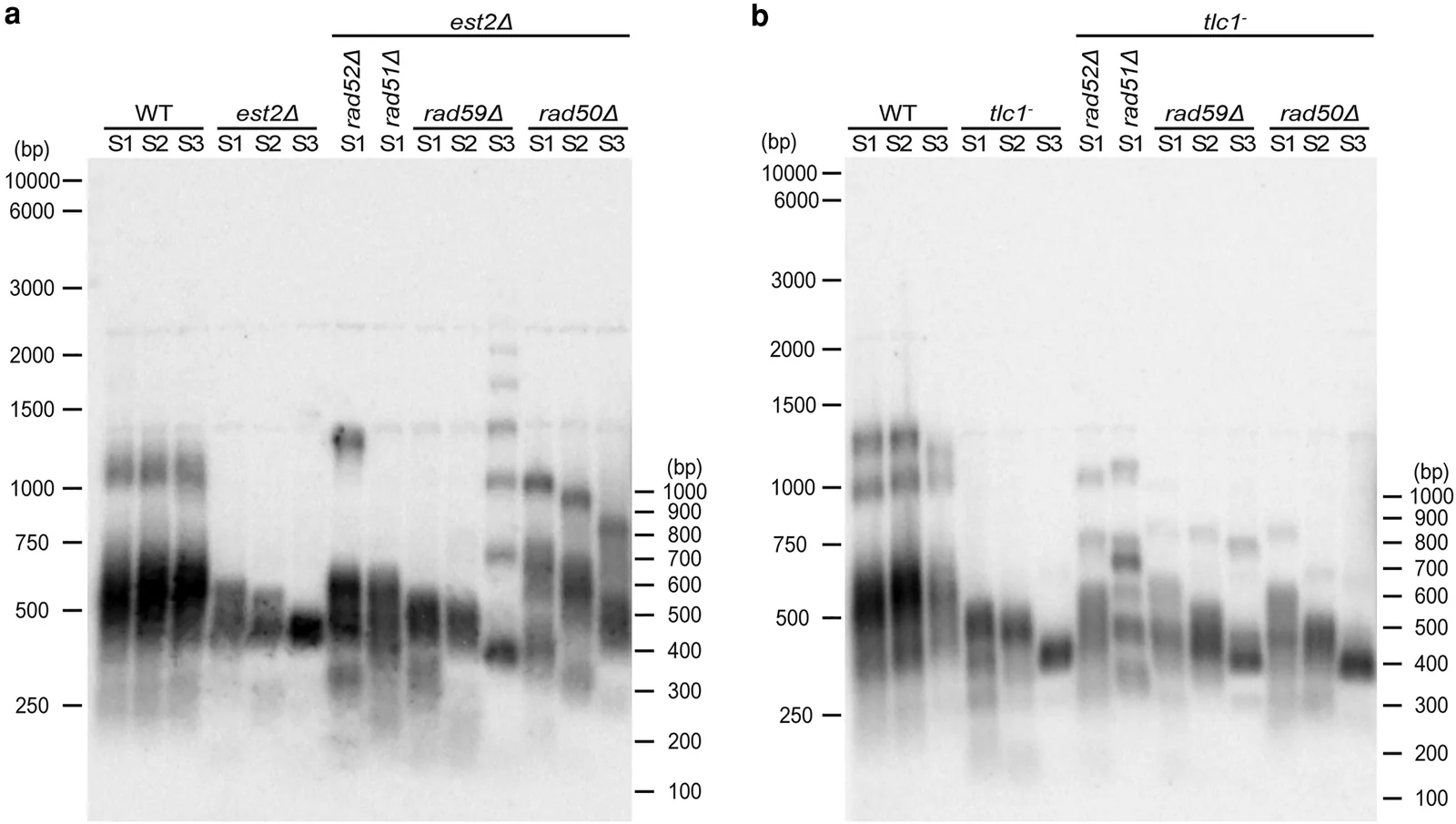
The telomere structure is rapidly modified in *N. castellii* ALT cells, in a mechanism dependent on *RAD52* and *RAD51*. TRF assays of genomic DNA from the indicated streaks (S) were digested with *Hinf*I, and the Southern blot membrane was hybridized with a 16-mer telomeric sequence probe. a) *EST2* double-KO mutant strains. b) *TLC1* double-KO mutant strains.

To evaluate the effect of the recombination gene KO on the length and structure of the telomeres, we performed TRF assays of our double-KO mutant strains (Fig. 5; Supplementary Figs. 10 and 11). In agreement with the previous results, WT strains showed a main telomere smear with a mean length of ∼600 bp, and both telomerase-deficient strain lines (*est2Δ* and *tlc1^−^*) showed progressive telomere shortening in the serial streaking assay, leading to a band at ∼400 bp in S3. The telomerase-KO strains in combination with *rad50Δ* or *rad59Δ* exhibited the same kind of progressive telomere shortening trait as the solely telomerase-deficient strains, resulting in S3 cells displaying a narrower band below 500 bp (Fig. 5; Supplementary Figs. 10 and 11). Noteworthy, in the *est2Δ rad59Δ* strains, the telomere shortening led to the appearance of the characteristic ALT ladder pattern in the S3 cells.

For the telomerase-KO strains with either *RAD52* or *RAD51* deletions, genomic DNA extraction could only be performed directly after the germination of the tetrad spore (S1), as it was not possible to obtain growth of the strains at later streaks. Compared to the WT isogenic strain, these double-KO strains indeed showed telomere shortening and changes in the telomeric band pattern. It is however noteworthy that the telomeres did not shorten at a faster rate in these double-KO strains, compared to the solely telomerase-deficient S1 strains. The control single *rad51Δ* and *rad52Δ* strains did not show any growth problems, and the telomere lengths were not affected (Supplementary Fig. 12).

Taken together, these results show that the establishment of the ALT pathway in *N. castellii* depends on homologous recombination mediated by Rad52 and Rad51. Moreover, telomerase-deficient cells without the *RAD50* and *RAD59* gene function can maintain short telomere characteristic of the activation of the ALT mechanism, thus indicating that these genes are not required for the establishment of the pathway.

## Discussion

Understanding the genetic requirements that drive the development of different ALT pathways is essential for the development of cancer therapies that seek to prevent the immortalization of cancer cells by targeting telomerase (Sugarman et al. 2019). Here, we first investigated the phenotypes associated with the loss of telomerase activity in the budding yeast *N. castellii* by KO of the genes coding for the respective core components of the holoenzyme, *EST2* and *TLC1*. We found that these newly created strain lines are capable of sustaining long-term growth by rapidly activating the ALT mechanism (Fig. 1). Using a quantitative column streak senescence assay, we show that they do not exhibit any early growth crisis (Fig. 2). Furthermore, we demonstrate a genetic requirement for *RAD52* and *RAD51*, indicating that the establishment of the ALT mechanism in *N. castellii* requires homologous recombination mediated by Rad52 and Rad51 proteins (Figs. 2 and 3). Interestingly, we found no evidence supporting any requirement of *RAD59* or *RAD50* gene function, indicating that recombination mediated by short-sequence homology at the telomeric repeats might not be a functional pathway for the rescue of telomeres in *N. castellii* (Fig. 3).

The genome of the nonconventional budding yeast *N. castellii* has been sequenced, providing the tools for the identification of open reading frames for genes previously uncharacterized in this species (Kanehisa and Goto 2000). Here we identified the homologs of the *RAD50*, *RAD51*, and *RAD59* genes (Supplementary Fig. 8). Like the previously characterized *RAD52* gene, these 3 genes show a high sequence identity to the corresponding *S. cerevisiae* genes both on the amino acid and nucleotide levels (Itriago et al. 2023). Further characterization of the *RAD51* gene showed that its function in DNA damage repair during genotoxic stress is conserved in this species (Supplementary Fig. 9). This implies that the recombinational pathways in *N. castellii* are highly similar to those in *S. cerevisiae*, which could be expected due to their close phylogenetic relationship.

In this study, we developed new lines of telomerase-deficient strains (*est2Δ* and *tlc1^−^*), using a different parental background than before (Cohn et al. 2019; Jaiswal et al. 2025). We found that they behave similarly as the previously analyzed *tlc1^−^* strains, thus reinforcing that the ALT pathway is a stable and reproducible cellular process for telomere rescue in *N. castellii*. Sequential passaging of the respective *est2Δ* and *tlc1^−^* strains for over 320 generations revealed that they proliferate indefinitely without major growth crisis and maintain wild-type (WT) colony morphology (Fig. 1b). As previously reported, a slightly lower growth rate of *tlc1^−^* cells was seen in liquid media when compared to the isogenic WT strain (Cohn et al. 2019; Jaiswal et al. 2025). Here we additionally report that *est2Δ* strains passaged in liquid media similarly show only a slightly lower growth rate (Fig. 1c). Therefore, the results contrast to the growth crisis experienced by *S. cerevisiae* and *K. lactis* telomerase-deficient strains, where only a few survivor cells manage to revert the onset of replicative senescence phenotypes (Lundblad and Szostak 1989; Lundblad and Blackburn 1993; McEachern and Blackburn 1996).

To further investigate the establishment of the ALT mechanism in *N. castellii*, we evaluated the growth ability of mutant haploid strains passaged on nutritious medium, using the column streak senescence assay (Fig. 2a). Our data demonstrate that *N. castellii est2Δ* and *tlc1^−^* mutant haploid strains do not exhibit replicative senescence in the early generations of growth after the loss of telomerase (Fig. 3b to d). A lack of a prominent senescence phenotype has also been observed in *Candida albicans* and *Yarrowia lipolytica* telomerase-KO mutants, and recently it was shown that *S. cerevisiae* strains containing a Y’ element in every chromosomal end can also avoid severe growth crisis and sustain growth without the appearance of senescence phenotypes, possibly due to an increase in the efficiency of the type I ALT pathway (Steinberg-Neifach and Lue 2006; Kinsky et al. 2010; Grandin et al. 2020). In *N. castellii*, the establishment of ALT cells leads to a homogenization of the subtelomeric regions, where the telomere-adjacent TelKO element is spread to all chromosome ends (Jaiswal et al. 2025). Interestingly, the TelKO element is not very abundant in the WT strain (YMC48), since it is present as a single copy in a subset of the chromosome ends. We therefore hypothesize that its specific structure might aid in the efficient addition of this sequence at the telomeres. We have shown that the telomere-binding protein Rap1 can bind to the TelKO element with low affinity, which may provide a possibility for the incorporation of this subtelomeric region into the terminal chromatin structure, enabling the formation of a stabilizing and protective telomere cap (Jaiswal et al. 2025).

To investigate the genetic requirement of the ALT pathway in *N. castellii*, we created several deletion strains of the recombination genes *RAD52*, *RAD50*, *RAD51*, and *RAD59*. The severe growth impairment found in the column streak senescence assay for telomerase-deficient and *rad52Δ* double-KO mutants demonstrates that *RAD52* gene function is required for the establishment of ALT in *N. castellii* (Fig. 2b and e), indicating that Rad52-mediated recombination is necessary for rescuing short telomeres in the absence of telomerase. We found that *RAD51* gene function is also essential for ALT establishment, as telomerase-deficient and *rad51Δ* double-KO mutants are unable to sustain growth (Fig. 3), which is consistent with *S. cerevisiae* type I survivors (Chen et al. 2001). Indeed, the severe growth defect and very early replicative senescence phenotype exhibited by these double-KO mutants are in accordance with the accelerated senescence shown in the corresponding *S. cerevisiae* double-KO mutants (Fig. 4) (Claussin and Chang 2016). Thus, the ALT pathway in *N. castellii* has the same genetic basis as the *S. cerevisiae* type I pathway, which is a Rad51-dependent break-induced replication (BIR) mechanism. However, since *N. castellii* exhibits a more effective ALT establishment, we speculate that the structure of the *N. castellii* subtelomeric regions may provide a better substrate for the homology search, which is very likely further enhanced by the uniformity of the telomeric repeats. Moreover, consistent with previous reports for *S. cerevisiae,* the *N. castellii* double-KO mutants did not show any accelerated telomere shortening, indicating that the early senescence phenotype observed is not only due to the shortening of the telomeres (Fig. 5) (Chen et al. 2001; Claussin and Chang 2016). As previously suggested, the loss of other non–BIR-related Rad52-mediated activities may be responsible for the early replicative senescence, although we cannot exclude that the lack of growth in S2 is due to an accelerated telomere shortening that is nondetectable in these experiments (Claussin and Chang 2016). The fact that a single critically short telomere triggers the onset of presenescent activities in *S. cerevisiae*, where Rad52-mediated homology-directed repair (HDR) plays a key role, leads us to hypothesize that similar activities in *N. castellii* may be responsible for establishing the early and effective onset of ALT maintenance (Abdallah et al. 2009).

S. *cerevisiae* survivors recovered after passaging telomerase-deficient cells in liquid media are commonly type II survivors (Aguilera et al. 2022). However, type I survivors can outcompete the fast-growing type II survivors when the majority of the chromosome ends contain the Y’ element (Grandin et al. 2020). We did not observe a significant growth defect in telomerase-deficient cells lacking *RAD50* or *RAD59* gene function (Fig. 3). Since the Rad50 and Rad59 proteins are essential in the Rad51-independent BIR pathway and given that we were unable to obtain survivor colonies of telomerase-deficient *rad51Δ* cells after 60 generations of growth, the data suggest that an ALT pathway relying on a Rad50/Rad59 repair mechanism, like in *S. cerevisiae* type II survivors (Chen et al. 2001), is not activated in *N. castellii* even in the absence of Rad51. This is interesting considering that *S. cerevisiae* type I strains containing Y’ elements in every chromosome can revert to type II in the absence of Rad51 (Grandin et al. 2020). Furthermore, we have not found any evidence for enrichment of the telomeric sequences in any of our telomerase-deficient strains. Although we cannot exclude the possibility that modest elongations might occur via recombination between telomeric sequences, our results suggest that it is not the primary mechanism of *N. castellii* ALT strains.

In *S. cerevisiae*, it was shown that recombination events between chromosomal ends containing Y’ elements are common in the early development of type I survivors (Kockler et al. 2021). This could also be happening in *N. castellii* cells and at accelerated rate due to the high efficiency of the pathway, leading to a rapid modification of the telomere structure (Fig. 5). Moreover, we hypothesize that in chromosome ends that lack the TelKO element, recombination could be mediated between the telomere region and the short interstitial telomeric sequence (ITS) that is copied during ALT (Cohn et al. 2019). Rap1 can bind this ITS in vitro (Jaiswal et al. 2025), and binding of Rap1 to subtelomeres could possibly stimulate homologous recombination by distorting the DNA conformation (Churikov et al. 2014). In *S. cerevisiae*, Rad59 facilitates the acquisition of Y’ elements in chromosomes lacking the sequence before the appearance of survivors, through replication at the TG-rich repeats (Churikov et al. 2014). Although we have not found any genetic requirement for *RAD50* and *RAD59*, other than the slightly lower growth capacities of the *RAD50* double-KO mutants (Fig. 4), we cannot discard the possibility that these proteins could play a role in spreading the TelKO element to telomeres that lack it as part of the mechanism.

Tandem repeats of the Y’ elements are commonly found at the chromosome ends of *S. cerevisiae* survivors, but the mechanism behind their formation has not been fully elucidated (Kockler et al. 2021). In *N. castellii*, the ladder patterns appearing in some streaks of the TRF assays visualize the addition of tandemly repeated composite elements, containing the subtelomeric TelKO element and telomeric sequences (Figs. 2d and 5) (Jaiswal et al. 2025). Given the efficiency of the pathway, it is possible that the generation of tandem repeats occurs through multiple recombination events at the same chromosomal end. The previous genome sequencing of an ALT strain revealed the occurrence of subtelomeric homogenization during ALT establishment, where long arrays of identical composite TelKO repeats are present at all the chromosome ends (Jaiswal et al. 2025). Such spreading of identical repeat arrays to all ends suggests the action of the roll-and-spread mechanism, involving ECC DNA (Natarajan and McEachern 2002; Xu and McEachern 2012). We note that the spreading is remarkably fast, as shown by the manifestation of a distinct band in the TRF assay already at 60 to 80 generations (S3 to S4) after the telomerase KO event. This is consistent with the rapid spreading of telomeric repeats observed in *K. lactis*, where sequences from a single source are spread to other telomeres (Natarajan and McEachern 2002; Topcu et al. 2005; Xu and McEachern 2012). We speculate that the excision of tandem subtelomeric repeats could lead to the formation of extrachromosomal circles that would act as templates for the roll-and-spread amplification. Such excisions could possibly cause the disappearance of the ladder pattern in the TRF assay (Fig. 1d, lanes S10 and S12). Therefore, it would be interesting to study the presence of such genetic elements in *N. castellii*, given that t-circles have been found in *S. cerevisiae* (Larrivee and Wellinger 2006; Aguilera et al. 2022).

An interesting observation, in this regard, is the rather late appearance of the ladder pattern, starting at S8 in the TRF assay (Fig. 1). The delayed elongation is a feature commonly seen in our strains, but the exact time of its appearance varies between different serial streaking assays. Although we cannot exclude that undetectable amounts of long arrays might be present also in the early streaks, the striking feature is that of the telomeric smear that is not only progressively shortening, but at the same time sharpening into a defined band. This is a general characteristic of all the telomerase-deficient strains, with the defined band generally occurring already at S3 to S4 (60 to 80 generations). This indicates that the subtelomeric homogenization precedes the generation of long arrays. In line with previous data showing that Rad52 is acting on the first critically short telomere (Abdallah et al. 2009), our data presented here implies that Rad52 and Rad51 are essential for the early events in the establishment of the ALT pathway. This leads us to hypothesize that these proteins may be the key players responsible for the primary recombination activities, laying the foundation for the creation and spreading of a specific composite TelKO element to all chromosome ends. The highly effective switch to the ALT pathway suggests that *N. castellii* telomeres are structurally predisposed for these recombinational activities, and it will be interesting to further investigate the underlying molecular mechanisms.

## Data Availability

The authors affirm that all data necessary for confirming the conclusions of the article are present within the article, figures, and tables. Materials and strains are available upon request. Supplementary data to this article can be found at FigShare (10.25387/g3.32414622). Supplemental material available at *G3* online.
